# Fat Body Mass and Vertebral Fracture Progression in Women With Breast Cancer

**DOI:** 10.1001/jamanetworkopen.2023.50950

**Published:** 2024-01-10

**Authors:** Deborah Cosentini, Rebecca Pedersini, Pierluigi Di Mauro, Manuel Zamparini, Greta Schivardi, Luca Rinaudo, Nunzia Di Meo, Andrea Del Barba, Carlo Cappelli, Marta Laganà, Andrea Alberti, Maria Baronchelli, Greta Guerci, Lara Laini, Salvatore Grisanti, Edda Lucia Simoncini, Davide Farina, Gherardo Mazziotti, Alfredo Berruti

**Affiliations:** 1 Department of Medical and Surgical Specialties, Radiological Sciences and Public Health, Medical Oncology, University of Brescia, ASST Spedali Civili, Brescia, Italy; 2 SSVD Breast Unit, ASST Spedali Civili of Brescia, Brescia, Italy; 3 Tecnologie Avanzate, Turin, Italy; 4 Department of Medical and Surgical Specialties, Radiological Sciences and Public Health, Radiology, University of Brescia, ASST Spedali Civili, Brescia, Italy; 5 Department of Experimental Sciences, Unit of Endocrinology and Metabolism, University of Brescia, ASST Spedali Civili, Brescia, Italy; 6 Department of Biomedical Sciences, Humanitas University, Milan, Italy; 7 Endocrinology, Diabetology and Medical Andrology Unit, Metabolic Bone Diseases and Osteoporosis Section, IRCCS Humanitas Research Hospital, Rozzano, Milan, Italy

## Abstract

**Importance:**

Women with early breast cancer (EBC) exposed to aromatase inhibitors (AIs) may experience fragility fractures despite treatment with bone-active drugs. Risk factors for fractures in patients receiving AIs and denosumab have not been explored to date.

**Objectives:**

To evaluate whether an association exists between dual x-ray absorptiometry (DXA)–measured fat body mass (FBM) and vertebral fracture (VF) progression in postmenopausal women with EBC undergoing adjuvant therapy with AIs in combination with denosumab and to examine whether VF was associated with common risk factors for bone fracture and parameters of body composition other than FBM.

**Design, Setting, and Participants:**

For this prospective, single-center, cohort study, 237 patients with EBC who were undergoing adjuvant treatment with AIs and denosumab (60 mg every 6 months) were enrolled at the Breast Unit of the ASST Spedali Civili of Brescia from September 2014 to June 2018. Data analysis was conducted in June 2022.

**Exposure:**

Body composition parameters, bone mineral density, and morphometric VFs were assessed by DXA at study entry and after 18 months of therapy.

**Main Outcomes and Measures:**

VF progression, defined as either new or worsening of preexisting VFs, between the 2 time points.

**Results:**

Of the 237 patients enrolled (median [range] age, 61 [28-84] years), 17 (4.4%) reported VF progression. Univariable analysis found an association between VF progression and a history of clinical fractures (odds ratio [OR], 3.22; 95% CI, 1.19-8.74; *P* = .02), Fracture Risk Assessment Tool (FRAX) score for major fractures (OR, 4.42; 95% CI, 1.23-13.79; *P* = .04), percentage of FBM (OR, 6.04; 95% CI, 1.69-21.63; *P* = .006), and android fat (OR, 9.58; 95% CI, 1.17-78.21; *P* = .04) and an inverse association with appendicular lean mass index–FBM ratio (OR, 0.25, 95% CI, 0.08-0.82; *P* = .02). Multivariable analysis revealed percentage of FBM (OR, 5.41; 95% CI, 1.49-19.59; *P* = .01) and FRAX score (OR, 3.95; 95% CI, 1.09-14.39; *P* = .04) as independent variables associated with VF progression.

**Conclusions and Relevance:**

The findings of this study suggest that baseline FBM is an independent factor for VF progression in patients with EBC treated with adjuvant AIs and denosumab. This observation is new and indicates that diet and exercise may synergize with denosumab in the management of bone health in this patient setting.

## Key Points

**Question:**

What are the risk factors for vertebral fracture progression in postmenopausal women with breast cancer undergoing adjuvant therapy with aromatase inhibitors and denosumab?

**Findings:**

In this cohort study of 237 patients, high baseline (ie, greater than the median) fat body mass and Fracture Risk Assessment Tool score for major fractures were independently associated with vertebral fracture progression at 18 months of adjuvant therapy with aromatase inhibitors and denosumab.

**Meaning:**

These findings suggest that fat body mass may promote skeletal fragility in postmenopausal women undergoing adjuvant aromatase inhibitors, despite the protective role of denosumab.

## Teaser

This cohort study evaluates whether fat body mass is associated with vertebral fracture progression in postmenopausal women with early breast cancer who are undergoing adjuvant therapy with aromatase inhibitors and denosumab.

## Introduction

Breast cancer is the most common cancer in women worldwide.^1,2^ Nearly 80% of early breast cancers (EBCs) in postmenopausal women are hormone receptor (HR)–positive^3^; 5-year adjuvant treatment with aromatase inhibitors (AIs) is the gold standard therapy^4^ for such patients. AIs deplete serum estrogen, leading to a marked increase in bone resorption,^5^ accompanied by a decrease in bone mineral density (BMD),^6,7,8^ ultimately increasing the risk of bone fracture.^8,9^

Current international guidelines for cancer treatment–induced bone loss^10,11^ recommend BMD as assessed by dual x-ray absorptiometry (DXA) and the Fracture Risk Assessment Tool (FRAX) score^12^ for determining baseline fracture risk in patients undergoing treatment with AIs. Both parameters are validated in postmenopausal osteoporosis but not in patients being treated with AIs.^13,14^

Recent studies have questioned the role of BMD in predicting fracture risk in women with EBC undergoing AI therapy.^15,16,17,18,19,20^ The limited role of BMD, a measure of bone quantity, in predicting the fracture risk in women undergoing AI therapy implies a predominant alteration of bone quality. Our group has developed the hypothesis that fat body mass (FBM) can contribute to bone quality deterioration during AI therapy^21^ via several mechanisms, including increased levels of inflammation cytokines and hormones (eg, adiponectin, leptin, insulin, parathyroid hormone) and decreased levels of vitamin D.^22,23,24^ Consistent with this hypothesis, our group conducted a cross-sectional study^18^ that found, for the first time of which we are aware, an association between FBM and a lower proportion of fractures in AI-naive and a higher fracture risk in AI-treated patients. This observation suggests that, unlike postmenopausal osteoporosis, high BMI and FBM may be related to the risk of fracture in women treated with AIs.

Of note is that denosumab, a bone resorption inhibitor, is increasingly prescribed in patients with EBC receiving AIs based on the results of the ABCSG-18 study.^25^ To our knowledge, fracture risk in patients with EBC receiving both AIs and denosumab has not been explored. We conducted a prospective, longitudinal cohort study to evaluate whether an association exists between DXA parameters of body composition, common risk factors for bone fracture, and progression of morphometric vertebral fracture (VF) in postmenopausal patients with EBC undergoing adjuvant therapy with AIs plus denosumab.

## Methods

This single-center cohort study was conducted at the Breast Unit of ASST Spedali Civili of Brescia from September 2014 to June 2018. The database was locked in June 2020, but data analysis was performed in June 2022 due to the COVID-19 pandemic. All patients received adjuvant hormonal therapy with AIs combined with denosumab (60 mg subcutaneous every 6 months) administered in a day hospital regime by the nursing staff. The ethics committee of ASST Spedali Civili of Brescia approved the study protocol and the informed consent forms according to the tenets of the Declaration of Helsinki.^26^ The study followed the Strengthening the Reporting of Observational Studies in Epidemiology (STROBE) reporting guideline.^27^

The primary objective was to determine whether an association exists between DXA-measured FBM (expressed in grams and as a percentage) and VF progression after AI therapy in combination with denosumab. The secondary objectives were (1) to determine whether an association exists between progression of VF during combined treatment with AIs and denosumab and common risk factors for bone fracture, as assessed by the FRAX tool and DXA-derived parameters of body composition other than FBM, ie, lean body mass (LBM in grams), appendicular lean mass index (ALMI, calculated as the sum of lean tissue in the arms and legs and then scaled to height squared [ALMI kg/m^2^]), an ALMI-FBM ratio, trunk appendicular fat ratio, total lean mass to height squared (LBM/h^2^), and total fat mass adjusted for height (FMI) and (2) to assess whether an association exists between VF at baseline conditions and the previously mentioned risk factors. As an explorative study, we evaluated the association between incident VF and android fat percentage, gynoid fat percentage, and android/gynoid ratio in a patient subset.

Key eligibility criteria were (1) histologically confirmed EBC, (2) eligibility for adjuvant treatment with AIs and denosumab, and (3) having signed the consent form. Previous chemotherapy was allowed but previous tamoxifen was not. Patients with poor performance status (Eastern Cooperative Oncology Group score ≥2), poor adherence (ie, not motivated to follow the study activities, difficulty traveling to our hospital, comorbidities, and/or lack of data on family history of fractures and on personal lifestyle), and previous treatment for other tumors were excluded. According to national^28,29^ and international^10,11^ guidelines, denosumab was prescribed in patients with a T score of less than −2 or any 2 of the following risk factors: older than 65 years, T score less than −1.5 SD, tobacco use (current and history of smoking), family history of either VF or hip fracture, personal history of fragility fracture after age 50 years, or oral glucocorticoid use for more than 6 months. The drug was prescribed on an individual basis in those with a T score greater than −1.5 SD, and there were no coexistent common risk factors for fracture, due to controversy in defining the therapeutic threshold in these specific conditions.^10,11,13,20,30^ DXA measurement was performed at baseline and again at 18 months by means of Hologic QDR-4500W instrumentation (Hologic Corporation). Two endocrinologists (A.D.B. and C.C.) and 2 radiologists (N.D.M. and D.F.), who were blinded to the clinical data, performed quantitative morphometric analysis of the DXA images.^31^ The fractures were classified as mild (height ratio decrease of 20%-25%), moderate (decrease of 26%-40%), or severe (decrease >40%). Discordant cases were resolved by consensus.

VF progression was defined as either new fracture (from no VF to any grade of VF) or worsening of preexisting VF (from mild to moderate or severe; from moderate to severe) between baseline and follow-up. The spine deformity index (SDI) was calculated by summing the grade of each vertebra from T4 to L4.^32^ Osteoporosis and osteopenia were defined according to World Health Organization criteria.^29^

Tobacco use was categorized as previous and current smoker or never smoked; alcohol use as greater or less than 12 g/d; physical activity as mild, moderate, or intense physical exercise; family history of fracture was defined as history of osteoporotic fracture in at least 1 first-degree relative. BMD was measured as gram per centimeters squared, and the T score was categorized as normal (>−1.0 SD) or within the range of osteopenia (−1.0 to −2.5 SD) or osteoporosis (≤−2.5 SD). In addition, we explored the association between baseline parameters and baseline SDI as well as changes in SDI at 18 months of treatment with AIs and denosumab.

### Statistical Analysis

A sample size was not calculated because of the exploratory nature of this study. Patient characteristics are presented as descriptive statistics. To test the potential association between the variables and morphometric VF, we estimated the odds ratios (ORs) using univariable logistic regression models. The results are expressed as ORs with 95% CIs.

Independent variables that showed a significant association (*P* < .10) with the dependent variable at univariable analysis were included in the multivariable model, except for variables that were measured in the patient subset. Using this approach, we derived the multivariable models through a backward elimination method. All statistical tests were 2-tailed, and the maximum type I error was 5% for all tests. Statistical analysis was performed using SPSS version 23.0 (IBM Corp). The Bonferroni-Holm method was applied to mitigate the risk of type I error inflation.^33^ Sensitivity analysis using the modified Poisson regression model was performed to validate data robustness.

## Results

### Patient Characteristics

From September 2014 to June 2018, 258 consecutive White women with HR-positive EBC were assessed for eligibility, 15 of whom were excluded because of refusal or ineligibility (eFigure in Supplement 1). The remaining 243 met the eligibility criteria and entered the study, 6 of whom were subsequently excluded because they did not undergo the second DXA scan at 18 months. A total of 237 patients (median [range] age, 61 [28-84] years) were assessed for BMD, T score, and FRAX score, 229 of whom were assessed for body composition (ie, FBM, LBM, ALMI, ALMI/FBM, trunk appendicular fat ratio, LBM/h^2^, and FMI). The explorative analysis of a subset of 197 patients also evaluated android fat, gynoid fat, and android/gynoid ratio. All patients received the 3 doses of denosumab as planned. Table 1 presents the clinical characteristics and the DXA-derived bone and body composition measurements.

**Table 1.** Patient Characteristics

Characteristic	Patients, No. (%) (N = 237)
Age, median (range), y	61 (28-84)
BMI	
Median (range)	24.3 (15.6-45.8)
<25	133 (59.9)
≥25	104 (53.3)
Body weight, median (range), kg	63 (43-117)
History of clinical fracture	
Yes	66 (27.8)
No	171 (72.2)
Family history of bone fracture	
Yes	19 (8.0)
No	218 (92.0)
Tobacco use	
Yes	52 (21.9)
No	185 (78.1)
Physical activity	
Yes	53 (22.4)
No	184 (77.6)
Alcohol use	
Yes	50 (21.1)
No	187 (78.9)
Total BMD, median (range), g/cm^2^	1.0 (0.6-1.4)
DXA	
Normal	54 (22.8)
Osteopenia	105 (44.3)
Osteoporosis	78 (32.9)
FRAX score for major fractures, median (range)	6.2 (1.4-34.0)
Tumor stage, No. (%)	
T1	157 (66.2)
T2-3-4	80 (33.7)
Nodal stage	
N0	139 (58.6)
N1-2-3	98 (41.4)
Histological type	
Ductal	169 (71.3)
Lobular	41 (17.3)
Other	20 (8.4)
Grade	
G1	10 (4.2)
G2-3	225 (95.8)
*ERBB2* positivity	48 (20.3)
Neoadjuvant or adjuvant chemotherapy	101 (42.6)
Baseline FBM, median (range), g	22 772.6 (6851.1-60 268.7)
Baseline FBM, median (range), %	36.0 (14.0-53.6)
Baseline LBM, median (range), g	38 867.1 (29 358.2-60 891.9)
Baseline trunk appendicular fat ratio, median (range)	0.95 (0.44-1.98)
Baseline lean mass/m^2^, median (range)	15 (0-22)
Baseline ALMI, median (range)	6 (0-10)
FMI, median (range)	9 (0-25)
Baseline android fat, median (range), g	37 (13-54)
Baseline gynoid fat, median (range), g	40 (18-54)
Baseline android gynoid ratio, median (range)	0.91 (0.39-1.42)

Abbreviations: ALMI, appendicular lean mass index; BMI, body mass index (calculated as weight in kilograms divided by height in meters squared); BMD, bone mineral density; DXA, dual x-ray absorptiometry; FBM, fat body mass; FMI, total fat mass to height; FRAX, Fracture Risk Assessment Tool; LBM, lean body mass.

### Risk Factors Associated With Baseline VFs

Baseline assessment revealed morphometric vertebral fracture in 40 patients (16.9%). Univariable and multivariable logistic analysis of the association between clinical and DXA-derived risk factors and baseline morphometric vertebral fracture is presented in Table 2. Univariable analysis found an association between VF and age (OR, 2.34; 95% CI, 1.16-4.71), history of clinical fractures (OR, 5.54; 95% CI, 2.70-11.36), BMD less than the median (OR, 2.18; 95% CI, 1.06-4.53), DXA diagnosis of osteoporosis (OR, 2.76; 95% CI, 1.03-7.42), and FRAX score for major fractures greater than the median (OR, 10.10; 95% CI 3.46-29.49). Only history of clinical fractures (OR, 3.38; 95% CI, 1.55-7.35; *P* = .002) and FRAX score (OR, 7.26; 95% CI, 2.41-21.90, *P* < .001) were identified as independent variables at multivariable analysis.

**Table 2.** Factors Associated With Morphometric Vertebral Fracture at Baseline

Risk factor	Patients, No. (N = 237)	Vertebral fracture, No. (row %)		Univariable analysis		Multivariable analysis	
		No	Yes	OR (95% CI)	*P* value	OR (95% CI)	*P* value
Age							
<Median	130	115 (88.5)	15 (11.5)	1 [Reference]	.02	1 [Reference]	.82
≥Median	107	82 (76.6)	25 (23.4)	2.34 (1.16-4.71)		0.91 (0.39-2.12)	
BMI							
<25	133	110 (82.7)	23 (17.3)	1 [Reference]	.85	NA	NA
≥25	104	87 (83.7)	17 (16.3)	0.94 (0.47-1.86)		NA	
History of bone fracture							
No	171	155 (90.6)	16 (9.4)	1 [Reference]	<.001^a^	1 [Reference]	.002^a^
Yes	66	42 (63.6)	24 (36.4)	5.54 (2.70-11.36)		3.38 (1.55-7.35)	
Tobacco use							
No	185	156 (84.3)	29 (15.7)	1 [Reference]	.35	NA	NA
Yes	52	41 (78.8)	11 (21.2)	1.44 (0.67-3.13)		NA	
Physical activity							
No	184	155 (84.2)	29 (15.8)	1 [Reference]	.39	NA	NA
Yes	53	42 (79.2)	11 (20.8)	1.40 (0.65-3.03)		NA	
Alcohol use							
No	187	157 (84.0)	30 (16.0)	1 [Reference]	.51	NA	NA
Yes	50	40 (80.0)	10 (20.0)	1.31 (0.59-2.90)		NA	
Family history of bone fracture							
No	218	184 (84.4)	34 (15.6)	1 [Reference]	.08	1 [Reference]	.75
Yes	19	13 (68.4)	6 (31.6)	2.50 (0.89-7.03)		1.21 (0.37-3.97)	
BMD total							
<Median	116	91 (78.4)	25 (21.6)	2.18 (1.06-4.53)	.04	1.10 (0.50-2.50)	.80
≥Median	121	106 (87.6)	15 (12.4)	1 [Reference]		1 [Reference]	
DXA T score							
Normal	54	48 (88.9)	6 (11.1)	1 [Reference]	NA	NA	NA
Osteopenia	105	91 (86.7)	14 (13.3)	1.23 (0.45-3.41)	.69	NA	NA
Osteoporosis	78	58 (74.4)	20 (25.6)	2.76 (1.03-7.42)	.04	NA	NA
FRAX score for major fractures							
<Median	110	106 (96.4)	4 (3.6)	1 [Reference]	<.001^a^	1 [Reference]	<.001^a^
≥Median	127	91 (71.7%)	36 (28.3%)	10.10 (3.46-29.49)		7.26 (2.41-21.90)	
FBM							
<Median	121	100 (82.6)	21 (17.4)	1 [Reference]	.92	NA	NA
≥Median	108	90 (83.2)	18 (16.8)	0.92 (0.48-1.93)		NA	
% of FBM							
<Median	122	100 (82.0)	22 (18.0)	1 [Reference]	.69	NA	NA
≥Median	107	90 (84.0)	17 (16.0)	0.89 (0.44-1.74)		NA	
LBM							
<Median	115	99 (86.1)	16 (13.9)	1 [Reference]	.24	NA	NA
≥Median	114	91 (79.8)	23 (20.2)	1.53 (0.76-3.10)		NA	
ALMI/FBM							
<Median	99	81 (81.8)	18 (18.2)	1 [Reference]	.56	NA	NA
≥Median	130	109 (85.8)	21 (16.2)	0.81 (0.40-1.64)		NA	
Trunk appendicular fat ratio							
<Median	121	104 (86.0)	17 (14.0)	1 [Reference]	.19	NA	NA
≥Median	108	86 (79.6)	22 (20.4)	1.61 (0.79-3.29)		NA	
Lean mass/h^2^							
<Median	118	98 (83.1)	20 (16.9)	1 [Reference]	.99	NA	NA
≥Median	111	92 (82.9)	19 (17.1)	1.00 (0.51-2.00)		NA	
ALMI							
<Median	112	95 (84.8)	17 (15.2)	1 [Reference]	.47	NA	NA
≥Median	117	95 (81.2)	22 (18.8)	1.29 (0.65-2.59)		NA	
FMI							
<Median	119	98 (82.2)	21 (17.8)	1 [Reference]	.80	NA	NA
≥Median	110	92 (83.5)	18 (16.5)	0.91 (0.46-1.82)		NA	
Android fat^b^							
<Median	98	82 (83.7)	16 (16.3)	1 [Reference]	.59	NA	NA
≥Median	89	77 (86.5)	12 (13.5)	0.80 (0.36-1.80)		NA	
Gynoid fat^b^							
<Median	96	80 (83.3)	16 (16.7)	1 [Reference]	.51	NA	NA
≥Median	91	79 (86.8)	12 (13.2)	0.76 (0.34-1.71)		NA	
Android-gynoid ratio^b^							
<Median	98	84 (85.7)	14 (14.3)	1 [Reference]	.78	NA	NA
≥Median	89	75 (84.3)	14 (15.7)	1.12 (0.50-2.50)		NA	

Abbreviations: ALMI, appendicular lean mass index; BMI, body mass index (calculated as weight in kilograms divided by height in meters squared); BMD, bone mineral density; DXA, dual x-ray absorptiometry; FBM, fat body mass; FMI, total fat mass to height; FRAX, Fracture Risk Assessment Tool; LBM, lean body mass; NA, not applicable; OR, odds ratio.

^a^ Retained statistical significance after Bonferroni-Holm post hoc correction.

^b^ These analyses were conducted in a subset with 187 patients.

There was an association between an SDI of 2 or greater at baseline and history of clinical fracture (OR, 4.33; 95% CI, 1.92- 9.77), family history of bone fracture (OR, 4.09; 95% CI, 1.41-11.84), and FRAX score (OR, 13.38; 95% CI, 3.09- 57.93) at univariable analysis. Multivariable analysis showed an independent association with history of clinical fracture (OR, 2.43; 95% CI, 1.02- 5.75; *P* = .04) and FRAX score (OR, 10.16; 95% CI, 2.28- 45.26; *P* = .004) (eTable 1 in Supplement 2).

### Progression of VFs and SDI After Therapy With AI and Denosumab

**Figure 1.**
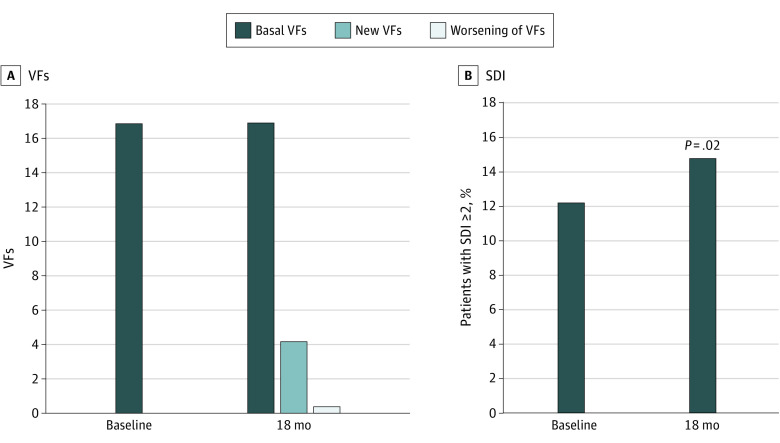
Percentage of Patients With Vertebral Fractures (VFs) and Spine Deformity Index (SDI) of 2 or Greater at Baseline and 18 Months of Treatment With Aromatase Inhibitors and Denosumab

Vertebral fracture was noted in 40 patients (16.9%) at baseline and in 50 (21.3%) at 18 months of AI treatment (*P* = .002). Progression of VF (new fractures and worsening of fracture grade) was noted in 17 patients (4.4%) (Figure 1A). The proportion of patients with an SDI of 2 or greater was 12.2% at baseline and 14.8% at 18 months (*P* = .02) (Figure 1B).

### Risk Factors Associated With Progression of VFs

**Figure 2.**
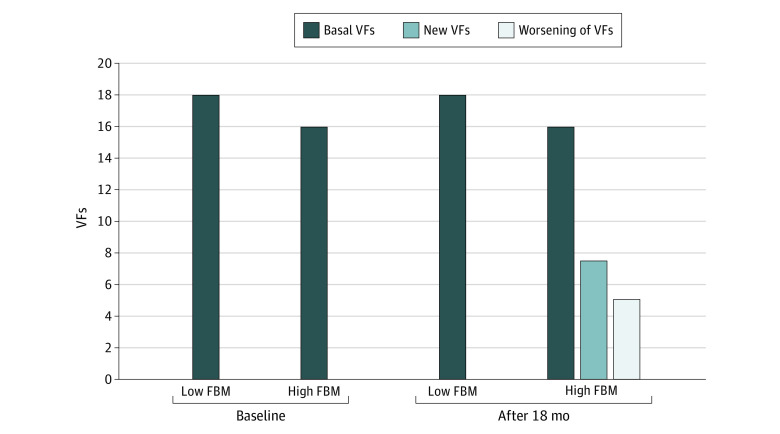
Percentage of Patients With Vertebral Fractures (VFs) at Baseline and New or Worsening VFs at 18 Months of Therapy, Stratified by Low and High Fat Body Mass (FBM) Low FBM defined as less than the median; high FBM as the median or greater.

Table 3 presents the association between progression of VF, clinical characteristics, and DXA-derived parameters. Univariable analysis found an association between vertebral fracture progression and history of clinical fractures (OR, 3.22; 95% CI, 1.19-8.74), FRAX score (OR, 4.42; 95% CI, 1.23-13.79), percentage of FBM (OR, 6.04; 95% CI, 1.69-21.63), android fat (OR, 9.58; 95% CI, 1.17-78.21), and ALMI/FBM ratio (OR, 0.25; 95% CI, 0.08-0.82). Multivariable analysis revealed an independent association between FRAX score (OR, 3.95; 95% CI, 1.09-14.39; *P* = .04) and percentage of FBM (OR, 5.41; 95% CI, 1.49-19.59; *P* = .01) and VF progression. Since android fat was evaluated in a patient subset, this parameter was not included in the multivariable model. Sensitivity analysis with modified Poisson regression (eTable 2 in Supplement 2) confirmed the logistic regression analysis. When the patients were stratified by percentage of FBM less or greater than the median, 2% with progression of VF after adjuvant AIs plus denosumab therapy had low FBM and 12.5% had high FBM (Figure 2). There was an association between worsening of SDI and history of clinical fractures (OR, 4.25; 95% CI, 1.16- 15.57) and baseline percentage of FBM (OR, 11.13; 95% CI, 1.39- 89.41); multivariable analysis showed that both factors were independently associated (history of clinical fractures: OR, 4.39; 95% CI 1.16- 16.63; *P* = .03; percentage of FBM: OR, 11.29; 95% CI, 1.39- 91.69; *P* = .02) (eTable 3 in Supplement 2).

**Table 3.** Baseline Risk Factors for Progression of Morphometric Vertebral Fracture After Adjuvant Treatment With Aromatase Inhibitors and Denosumab

Risk factor	Patients (N = 237)	Patients with fracture progression, No. (row %)		Univariable analysis		Multivariable analysis	
		No	Yes	OR (95% CI)	*P* value	OR (95% CI)	*P* value
Age							
<Median	130	120 (92.3)	10 (7.7)	1 [Reference]	.73	NA	NA
≥Median	107	100 (92.5)	7 (6.5)	0.84 (0.31-2.29)		NA	
BMI							
<25	133	125 (94.0)	8 (6.0)	1 [Reference]	.44	NA	NA
≥25	104	95 (91.3)	9 (8.7)	1.48 (0.55-3.98)		NA	
History of clinical fractures							
No	171	163 (95.3)	8 (4.7%)	1 [Reference]	.02	1 [Reference]	.15
Yes	66	57 (86.4)	9 (13.6)	3.22 (1.19-8.74)		2.29 (.73-7.15)	
Tobacco use							
No	185	173 (93.5)	12 (6.5)	1 [Reference]	.44	NA	NA
Yes	52	47 (90.4)	5 (9.6)	1.53 (0.52-4.57)		NA	
Physical activity							
No	184	171 (92.9)	13 (7.1)	1 [Reference]	.91	NA	NA
Yes	53	49 (92.5)	4 (7.5)	1.07 (0.34-3.44)		NA	
Alcohol use							
No	187	174 (93.0)	13 (7.0)	1 [Reference]	.80	NA	NA
Yes	50	46 (92.0)	4 (8.0)	1.16 (0.36-3.74)		NA	
Family history of bone fracture							
No	218	204 (93.6)	14 (6.4)	1 [Reference]	.14	NA	NA
Yes	19	16 (84.2)	3 (15.8)	2.73 (0.71-10.51)		NA	
BMD total							
<Median	116	105 (91.5)	11 (9.5)	2.10 (6.34-0.69)	.19	NA	NA
≥Median	121	115 (95.0)	6 (5.0)	1 [Reference]		NA	
DXA T score							
Normal	54	52 (96.3)	2 (3.7)	1 [Reference]	.45	NA	NA
Osteopenia	105	100 (95.2)	5 (4.8)	1.30 (0.24-6.93)	.09	NA	NA
Osteoporosis	78	68 (87.2)	10 (12.8)	3.82 (0.80-18.21)	NA	NA	NA
FRAX							
<Median	110	107 (97.2)	3 (2.8)	1 [Reference]	.04	1 [Reference]	.04^a^
≥Median	127	113 (89.0)	14 (11.0)	4.42 (1.23-13.79)		3.95 (1.09- 14.39)	
FBM							
<Median	121	115 (95.0)	6 (5.0)	1 [Reference]	.14	NA	NA
≥Median	108	97 (89.7)	11 (10.3)	2.20 (0.78-6.16)		NA	
% of FBM							
<Median	122	119 (97.6%)	3 (2.4%)	1 [Reference]	.006^a^	1 [Reference]	.01^a^
≥Median	107	93 (86.8)	14 (13.2)	6.04 (1.69-21.63)		5.41 (1.49- 19.59)	
LBM							
<Median	115	105 (91.3)	10 (8.7)	1 [Reference]	.21	NA	NA
≥Median	114	107 (93.9)	7 (6.1)	0.50 (0.16-1.50)		NA	
ALMI/FBM ratio							
<Median	99	87 (86.2)	12 (13.8)	1 [Reference]	.02	1 [Reference]	.45
≥Median	130	125 (96.2)	5 (3.8)	0.25 (0.08-0.82)		0.53 (0.10-2.76)	
Trunk appendicular fat ratio							
<Median	121	113 (93.4)	8 (6.6)	1 [Reference]	.61	NA	NA
≥Median	108	99 (91.7)	9 (8.3)	1.34 (0.44-4.14)		NA	
Lean mass/h^2^							
<Median	118	107 (90.7)	11 (9.3)	1 [Reference]	.23	NA	NA
≥Median	111	105 (94.6)	6 (5.4)	0.51 (0.18-1.53)		NA	
ALMI							
<Median	112	103 (91.3)	9 (8.7)	1 [Reference]	.72	NA	NA
≥Median	117	109 (93.2)	8 (6.8)	.83 (0.29-2.36)		NA	
FMI							
<Median	119	113 (95.0)	6 (5.0)	1 [Reference]	.14	NA	NA
≥Median	110	99 (90.0)	11 (10.0)	2.28 (0.76-6.91)		NA	
Android fat^b^							
<Median	98	97 (99.0)	1 (1.0)	1 [Reference]	.04	NA	NA
≥Median	89	81 (91.0)	8 (9.0)	9.58 (1.17-78.21)		NA	
Gynoid fat^b^							
<Median	96	93 (96.9)	3 (3.1)	1 [Reference]	.28	NA	NA
≥Median	91	85 (93.4)	6 (6.6)	2.19 (0.53-9.02)		NA	
Android gynoid ratio^b^							
<Median	98	96 (98.0)	2 (2.0)	1 [Reference]	.08	NA	NA
≥Median	89	82 (92.1)	7 (7.9)	4.10 (0.83-20.27)		NA	

Abbreviations: ALMI, appendicular lean mass index; BMI, body mass index (calculated as weight in kilograms divided by height in meters squared); BMD, bone mineral density; DXA, dual x-ray absorptiometry; FBM, fat body mass; FMI, total fat mass to height; FRAX, Fracture Risk Assessment Tool; LBM, lean body mass; NA, not applicable; OR, odds ratio.

^a^ Retained statistical significance after Bonferroni-Holm post hoc correction.

^b^ The variables android fat, gynoid fat, and android-gynoid ratio were not included in the multivariable model because they were assessed in a subset of 187 patients.

## Discussion

Bone resorption inhibitors are frequently administered to patients with EBC receiving adjuvant AIs.^25,34,35^ This prospective study was designed to explore the risk factors for progression of VF in patients with EBC receiving AIs and denosumab. The results found an association between adiposity and VF progression, also after correction for common risk factors of fracture, as assessed with the FRAX tool. These data contrast with those reported for postmenopausal women, in which obesity often plays a protective role.^36^ American Society of Clinical Oncology and European Society for Medical Oncology guidelines on bone health in patients with cancer state that high BMI is a low risk factor for bone fracture in postmenopausal women undergoing AI treatment.^10,11^ Our data, however, raise doubt about current guideline recommendations for the management of survivors with EBC.^10,11^

The complex relationship between obesity and bone fragility^37^ resides in the balance between 2 contending mechanisms: (1) protective via increased estrogen levels that increase BMD and (2) detrimental via the production of inflammatory proteins and other endocrine and paracrine factors that alter bone quality. These 2 contrasting actions form the so-called paradox of obesity.^23,37^ Under conditions beyond the context of patients with EBC treated with AIs, the effect of estrogens generally prevails so that the fracture risk in women with obesity is usually low. When a patient with overweight or obesity receives AI treatment, however, the reduction in BMD due to estrogen deprivation synergizes with the negative action of adiposity on bone quality.^13,18^ As a consequence, patients with overweight or obesity protected at baseline encounter a high risk of bone fragility fracture during treatment with AIs.

The correlation between FBM and fracture risk in women treated with AIs was observed in a cross-sectional study by our group.^18^ The present study shares these results in a prospective series of patients and the observed association, also with the addition of denosumab, which exerts a favorable effect on bone quality^38^ and potential extraskeletal effects on body composition.^39^ Since RANK-L and RANK are distributed in both skeletal and extraskeletal tissue including fat tissue,^40^ it may be hypothesized that modulation of RANK-L by denosumab may induce long-term changes in bone-fat crosstalk, regardless of its beneficial effects on bone remodeling and skeletal health. From this point of view, the association between higher FBM and progression of VF in women treated with denosumab would have been more evident with other bone-active drugs (eg, bisphosphonates) that have not been shown to have a favorable effect on body composition.^39^

In addition to fat, muscle tissue also plays an important role in maintaining bone health. Strain induced by muscle contraction stimulates bone growth because osteoblasts and osteocytes are mechanosensitive.^41^ Moreover, muscle-secreted myokines (eg, interleukins, irisin, myostatin, growth factors) can regulate bone metabolism.^42^ In a small prospective study conducted by our group involving patients with prostate cancer treated with a luteinizing hormone-releasing hormone analogue antagonist, we found a strong correlation between ALMI, which is an expression of the muscle mass of the limbs, and C-terminal telopeptide of type I collagen at either baseline or after treatment.^43^ In a previous cross-sectional study of patients with EBC who were either AI-naive or AI-treated, we explored the interaction of FBM and LBM with fracture risk and observed that VF was more often associated with the low fat mass and the low lean mass phenotype in AI-naive women, whereas high FBM and low LBM were associated with a higher proportion of VF in AI-treated women.^17^

In the present study, the association between progression of VF and lean mass parameters was lost in the multivariable analysis; this suggests that LBM might play a less important role than FBM in this clinical setting. Indeed, we cannot rule out the possibility that denosumab’s positive action on muscle function might have mitigated the association between sarcopenia and fracture risk in women exposed to AIs.^39^ Therefore, our study leaves the question open whether the correlation between DXA parameters of sarcopenic obesity and fracture risk may be more evident in patients treated with bisphosphonates, which do not exert effects on body composition and adipose function.^39^ Finally, our results in patients with breast cancer might be more impressive in other clinical settings, such as prostate cancer, where sarcopenic obesity is frequent and clinically more relevant.^21^ These hypotheses merit future research.

As regards DXA-related bone parameters and clinical risk factors of bone fracture, we noted an independent association between FRAX score and history of clinical fractures and baseline or progression of VF. Previous studies questioned the role of the FRAX algorithm in predicting bone fracture in patients receiving hormone-deprivation therapies.^13^ It is possible that the therapeutic thresholds of FRAX score already in use may be adapted to the specific context of patients exposed to hormone-deprivation therapies.^13,20,44^ Differently, we found no association with BMD. These data point toward a limited role of BMD in evaluating AIs-induced risk of skeletal fragility fractures.^15,25^

### Strengths and Limitations

This study has strengths. This prospective single-center study involved patients evaluated with a single DXA instrument and by the same team of radiologists and endocrinologists.

However, this study also has limitations, mainly its exploratory nature and the relatively small number of patients. Our primary focus was to describe the association between risk factors and vertebral fracture after 18 months of therapy. We did not use a time-to-event regression model or a model accounting for competing risks, which may constitute a further limitation.

## Conclusions

This prospective study provides initial evidence for an association between FBM and VF progression in postmenopausal women undergoing adjuvant therapy with AIs, despite the protective effect of denosumab. These data deserve further study in a validation cohort and in a patient population treated with AIs without denosumab. Diet and exercise may positively synergize with denosumab in the management of bone health in patients with EBC receiving adjuvant AIs.

## Notes

Supplement 1. **eFigure.** Consort Diagram

Supplement 2. **eTable 1.** Risk Factors for Spine Deformity Index (SDI) of 2 or Greater at Baseline **eTable 2.** Baseline Risk Factors for Morphometric Vertebral Fracture Progression After Treatment With Adjuvant Aromatase Inhibitors (AIs) and Denosumab: Modified Poisson Regression for Dichotomous Outcomes **eTable 3.** Baseline Risk Factors for Worse Spine Deformity Index (SDI) at 18 Months of Treatment With Aromatase Inhibitors (AIs) and Denosumab

Supplement 3.
Nonauthor Collaborators

Supplement 4.
Data Sharing Statement
