# Error in Coauthor Name

**DOI:** 10.1001/jamanetworkopen.2024.2491

**Published:** 2024-02-15

In the Original Investigation titled “Fat Body Mass and Vertebral Fracture Progression in Women With Breast Cancer,”^1^ published January 10, 2024, there was an error in the spelling of a coauthor’s name. Andrea Del Barba, MD, should have appeared as Andrea Delbarba, MD. This article has been corrected.^1^

## References

[1] Cosentini D, Pedersini R, Di Mauro P, ; Bone Health Group of the ASST Spedali Civili, Brescia. Fat body mass and vertebral fracture progression in women with breast cancer. JAMA Netw Open. 2024;7(1):e2350950. doi:10.1001/jamanetworkopen.2023.50950 38198137 PMC10782249

