# Supplementary material for: Fat Body Mass and Vertebral Fracture Progression in Women With Breast Cancer
Source: JAMA Netw Open. 2024 Jan 10;7(1):e2350950. doi: 10.1001/jamanetworkopen.2023.50950 (PMC10782249; doi:10.1001/jamanetworkopen.2023.50950)
Supplement: Supplement 1. — eFigure. Consort Diagram [file jamanetwopen-e2350950-s001.pdf]

Supplemental Online Content

Cosentini D, Pedersini R, Di Mauro P, et al; the Bone Health Group of the ASST Spedali Civili, Brescia. Fat body mass and vertebral fracture progression in women with breast cancer. *JAMA Netw Open.* 2024;7(1):e2350950. doi:10.1001/jamanetworkopen.2023.50950

eFigure. Consort Diagram

This supplemental material has been provided by the authors to give readers additional information about their work.

© 2024 Cosentini D et al. *JAMA Network Open.*

eFigure. Consort Diagram

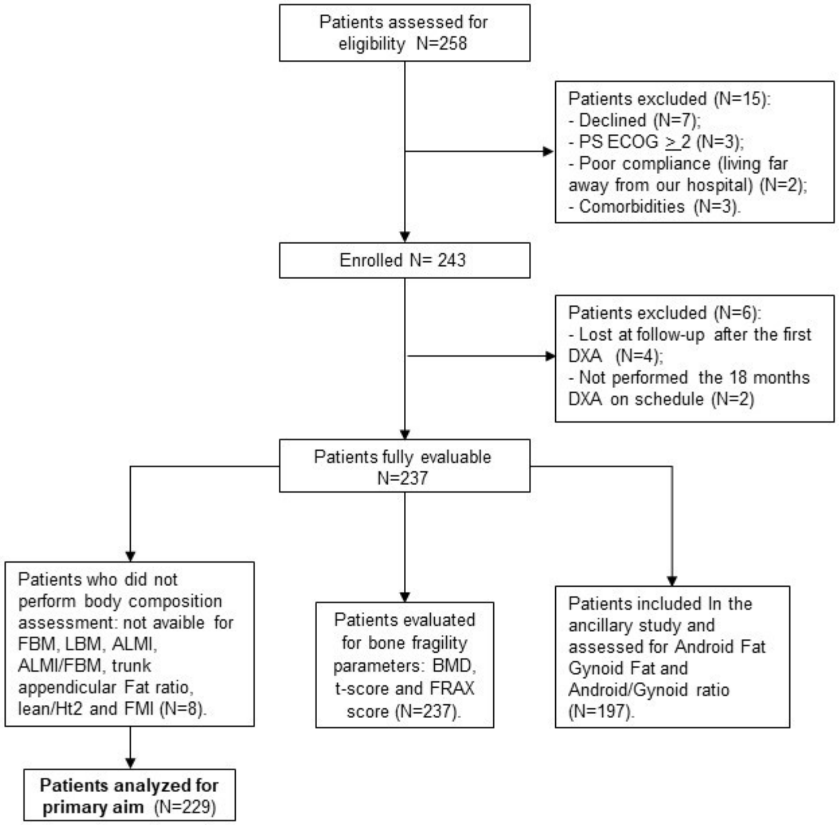

© 2024 Cosentini D et al. *JAMA Network Open.*
