# Supplementary material for: Fat Body Mass and Vertebral Fracture Progression in Women With Breast Cancer
Source: JAMA Netw Open. 2024 Jan 10;7(1):e2350950. doi: 10.1001/jamanetworkopen.2023.50950 (PMC10782249; doi:10.1001/jamanetworkopen.2023.50950)
Supplement: Supplement 3. — Nonauthor Collaborators [file jamanetwopen-e2350950-s003.pdf]

Supplemental Online Content: Nonauthor Collaborators
*First name, last name, and suffix (if applicable) are required and will appear in PubMed.

***Group Name(s): Bone health group of the ASST Spedali Civili of Brescia**

***First Name and Middle Initial(s)**	***Last Name**	***Suffix (eg, Jr, III)**	Academic Degrees	Institution	Location (city, state/province, country)	Role or Contribution, eg, chair, principal investigator	Group (if more than 1 Group listed in the byline) and/or Subgroup (eg, Steering Committee)
Monica	Boglioni	Dr	MD	ASST Spedali Civili	Brescia, Italy		Study coordinator
Giulia	Calzoni	Dr	MD	ASST Spedali Civili	Brescia, Italy		Study coordinator
