# Supplementary material for: Fat Body Mass and Vertebral Fracture Progression in Women With Breast Cancer
Source: JAMA Netw Open. 2024 Jan 10;7(1):e2350950. doi: 10.1001/jamanetworkopen.2023.50950 (PMC10782249; doi:10.1001/jamanetworkopen.2023.50950)
Supplement: Supplement 4. — Data Sharing Statement [file jamanetwopen-e2350950-s004.pdf]

Data Sharing Statement

Cosentini. Fat Body Mass and Vertebral Fracture Progression in Women With Breast Cancer. *JAMA Netw Open*. Published January 10, 2024. doi:10.1001/jamanetworkopen.2023.50950

Data
Data available: No

Additional Information
Explanation for why data not available: We will share the data only after formal request
